# Predicting success of intrauterine insemination using a clinically based scoring system

**DOI:** 10.1007/s00404-022-06758-z

**Published:** 2022-09-07

**Authors:** Anna Lena Zippl, Alfons Wachter, Patrick Rockenschaub, Bettina Toth, Beata Seeber

**Affiliations:** 1 Department of Gynecological Endocrinology and Reproductive Medicine, Medical University Innsbruck, Anichstr. 35, 6020 Innsbruck, Austria; 2 Institute of Health Informatics, University College London, London, NW1 2DA UK

**Keywords:** Infertility, IUI, Pregnancy rate, Prediction model, Clinical score

## Abstract

**Purpose:**

To develop a predictive score for the success of intrauterine insemination (IUI) based on clinical parameters.

**Methods:**

We performed a retrospective cohort study evaluating the homologous IUI cycles performed at a single university-based reproductive medical center between 2009 and 2017. The primary outcome measure was pregnancy, defined as positive serum human chorionic gonadotropin (hCG) 12–14 days after IUI. Predictive factors for pregnancy after IUI were identified, and a predictive score was developed using a multivariable continuation ratio model.

**Results:**

Overall, 1437 IUI cycles in 758 couples were evaluated. We found a per cycle pregnancy rate of 10.9% and a cumulative pregnancy rate of 19.4%. In a multivariable analysis, the probability of pregnancy was negatively associated with female age ≥ 35 years (OR 0.63, 95% CI 0.41–0.97, *p* = 0.034), endometriosis, unilateral tubal factor, or anatomical alteration (OR 0.54, 95% CI 0.33–0.89, *p* = 0.016), anti-Mullerian hormone (AMH) < 1 ng/ml (OR 0.50, 95% CI 0.29–0.87, *p* = 0.014), and total progressive motile sperm count (TPMSC) < 5 mil (OR 0.47, 95% CI 0.19–0.72, *p* = 0.004). We developed a predictive clinical score ranging from 0 to 5. Following 3 cycles, couples in our cohort with a score of 5 had a cumulative probability of achieving pregnancy of nearly 45%. In contrast, couples with a score of 0 had a cumulative probability of only 5%.

**Conclusion:**

IUI success rates vary widely depending on couples’ characteristics. A simple to use score could be used to estimate a couple’s chance of achieving pregnancy via IUI, facilitating individualized counseling and decision-making.

## What does this study add to the clinical work

**Table:** 

This study proposes a simple clinical score predicting a couple’s probability of achieving pregnancy via IUI, based on routinely obtained parameters. After external validation proving its generalizability, this model could facilitate patient-oriented counselling and decision making.	

## Introduction

Homologous intrauterine insemination (IUI) with or without ovarian stimulation has historically been used as first-line treatment for infertility. Today, due to increasing per cycle success rates of over 30% in achieving pregnancy through in vitro fertilization (IVF) [1], many couples choose this option as an initial treatment. In contrast, based on a multi-center retrospective analysis from 22 European centers evaluating more than 175,000 IUIs, IUI achieved a pregnancy rate of only 8.6% per cycle, albeit with large variability between centers ranging from 4 to 13%. Nonetheless, in women with unexplained infertility < 38 years, comparable live birth rates after ovarian stimulation with IUI and IVF have been reported [2]. As IUI is a relatively simple procedure associated with far lower risks and potential complications, and costs than IVF, it would be clinically useful to predict which patients have a high chance of pregnancy with IUI.

Although models predicting pregnancy after IUI have been previously published [3–7], they are not widely used in clinical routine practice. In fact, they are all based on semen analysis according to 1999 WHO criteria [8], and ovarian hyperstimulation aiming for two or three dominant follicles at ovulation induction (OI) prior to IUI. Considering the publication of new WHO criteria for semen analysis in 2010 [9] and the evidence showing that strict cancelation policies in case of multifollicular growth are effective in reducing the risk of multiple gestations following IUI [10–12], these models might be outdated. Recently, another predictive model has been published by Souter et al. [13] but its focus lies on ovarian stimulation and ovulation induction in infertility due to PCOS and unexplained infertility, not on IUI.

Based on these premises, we aimed to determine the per cycle pregnancy rate following IUI as well as the cumulative pregnancy rate after repeated cycles in a retrospective cohort of 758 couples undergoing 1437 IUIs between the years of 2009 and 2017 at our department. Our objective was to identify predictive factors for achieving pregnancy, including demographic and clinical data, infertility diagnosis and sperm parameters. We aimed to develop a simple clinical score based on these routinely obtained parameters, that would facilitate the counseling of infertile patients by providing evidenced-based individualized recommendations.

## Materials and methods

### Study population

After approval of the study protocol, data were collected retrospectively from 2009 to 2017 at the Department of Gynecological Endocrinology and Reproductive Medicine, Medical University Innsbruck, Austria. All homologous IUI treatments of women aged 18–45 years were included. Heterologous inseminations and discontinued treatment cycles (e.g., because of absence of follicular growth or multifollicular development with > 2 leading follicles) were excluded. Couples with severe male factor were not offered insemination and were not part of this study. We compared patient and cycle characteristics between those achieving a pregnancy and those who did not. In cases of multiple inseminations per patient during the period of observation, the first successful attempt or the last ineffective treatment cycle were included in the analysis, respectively.

### Procedures

All couples underwent a standardized diagnostic work-up including hormonal analysis throughout one cycle, sonographic and/or laparoscopic evaluation, and semen analysis according to 2010 WHO criteria [9]. Semen analysis followed the criteria published by Bjöhrndahl et al. [14] with few exceptions: For motility assessment, a disposable haemocytometer was used. We used Diff Quick (Giemsa staining) instead of Papanicolaou staining. Sperm vitality assessment was not part of the routine assessment before IUI, as in case of very low motility performance of an IUI is not appropriate independently of vitality. For similar reasons sperm antibodies were not examined. Following the standard protocol of our department, IUI was performed either after stimulation with clomiphene citrate or low-dose gonadotropins, or in the natural cycle, depending on female infertility diagnosis. Clomiphene treatment was chosen in case of PCOS, and started on or before the fifth day of the menstrual cycle with a standard dose of 50 mg per day for 5 days. Low-dose treatment with gonadotropins, chosen in case of hypothalamic anovulation or endometriosis, was started on the second or third day of the menstrual cycle in the dosage of 25–75 IU per day and was continued with a step-up protocol, as needed. In some patients with documented ovulatory menstrual cycles, IUI was performed after ultrasound confirmation of a leading follicle and ovulation induction (OI) in their natural cycle, without prior follicular stimulation, according to patients’ preference. Ovulation was induced using subcutaneously administered hCG (5.000–10.000 IU s.c.) when the leading follicle reached a size of ≥ 18 mm. Serum hormone evaluation for concentration of oestradiol, progesterone and luteinizing hormone (LH) was evaluated prior to trigger. Semen was collected on the day of insemination after two–seven days of sexual abstinence. Inseminations were carried out with a soft catheter 24–36 h after OI depending on LH and progesterone at time of trigger. All patients were supplemented with vaginal micronized progesterone (200 mg, three times daily) to support corpus luteum function.

### Outcome measures

The primary outcome measure was pregnancy, defined as positive serum hCG 12–14 days after IUI. IUI success was calculated as per cycle and cumulative probability of pregnancy.

### Covariates

Woman’s age, body mass index (BMI), and anti-Mullerian hormone (AMH) at the last included IUI cycle were extracted from the patient’s medical record. Female causes of infertility were retrospectively ascertained and categorized as anovulatory infertility (PCOS and hypothalamic amenorrhea), unilateral tubal factor, endometriosis, or anatomical alterations (uterine malformations, myoma). PCOS was defined according to the Rotterdam criteria [15]. A diagnosis of fallopian tube patency was based on hysterosalpingosonography (HSS) or chromopertubation. Cases of endometriosis were confirmed to be minimal to mild and treated with laparoscopy. Women whose work-up was negative were categorized as having no known female reason for infertility. Finally, total progressive motile sperm count (TPMSC) at the day of IUI was calculated using total sperm count and percentage of progressively motile sperm assessed prior to semen preparation via density gradient centrifugation. If any of the variables could not be obtained from the retrospective records, it was considered missing and imputed as described below.

### Statistical analysis

Patient characteristics were summarized using means and standard deviations (continuous variables) and counts and percentages (categorical variables). Differences between couples who did and did not achieve pregnancy were compared using *t* tests and χ^2^ tests. If continuous variables were found to be non-normally distributed, the median, interquartile range (IQR), and Wilcoxon rank-sum test were used instead. To investigate changes in pregnancy rate by number of cycles, we estimated the pregnancy rate per IUI cycle and calculated the cumulative probability of pregnancy. Assuming a constant pregnancy rate across cycles, expected 5% and 95% percentiles of observed pregnancies were calculated based on a Binomial distribution and compared to the observed number of pregnancies at each cycle. A change in time was formally tested using Fisher’s exact test.

The impact of patient characteristics on the probability of pregnancy at each cycle was modeled using a multivariable continuation ratio model, which uses logistic regression with a logit link to model each individual IUI cycle observed per couple [16]. IUI cycles entered the model as a linear covariate. Other covariates were assumed time-invariable. We considered different and increasingly complex models by first considering AMH (< 1 and ≥ 1 ng/ml), TMPSC (< 5, 5–15, and ≥ 15 mil) and female infertility diagnosis (anovulatory and unknown reasons for infertility versus all other diagnoses; Model 1), then substituting AMH by female age (< 35 versus ≥ 35 years; Model 2), and finally combining AMH and female age and further adding BMI (< 30 and ≥ 35 kg/m^2^; Model 3). Missing values were imputed using Multivariate Imputation by Chained Equations (MICE), resulting in 10 imputed datasets [17]. Model fit was compared using the Akaike Information Criterion (AIC), the Bayesian Information Criterion (BIC), and the c-index [16]. To account for optimism in the calculation of the c-index, optimism-adjusted bootstrap with 100 resamples was used [18]. The best fitting model was converted into a simple clinical score by rounding each covariate’s estimated coefficient to the closest multiple of a suitably picked denominator. Estimated cumulative success rates for each achievable score were calculated and provided graphically.

All analysis was performed using the R programming language (version 4.0.5) on Mac with the tidyverse (1.3.0), mice (3.13.0), and discSurv (1.4.1) packages. All code used for the analysis can be found at https://github.com/prockenschaub/IUI.

## Results

Between 2009 and 2017, 758 women aged 18–45 years (mean 33.4 ± 5.5; of these, 451 women < 35 years, 307 women ≥ 35 years) collectively underwent 1,437 IUI cycles (Table 1). Of the included couples, 255 (33.6%) suffered from female-related infertility, 152 (20.1%) from reduced male fertility, and 154 (20.3%) from both female and male infertility. The remaining 197 (26.0%) couples had infertility of unknown origin. Observed female aetiologies were: anovulatory infertility (26.9%), endometriosis (10.4%), unilateral tubal factor (8.2%) and anatomical alteration (5.5%). Most women were normal weight, with a median BMI of 22.3 kg/m^2^ (IQR 20.5, 25.8). Mean AMH was 2.40 ng/ml, with 160 (24.7%) women having an AMH < 1 ng/ml. In 51.1% of the analyzed IUI cycles, women underwent a low-dose stimulation with gonadotropins, while in 27.0% clomiphene was used (0.8% received both). In 21.1% of the cycles, IUI was performed in the natural cycle, without prior follicular stimulation. 306 (40.4%) men were diagnosed with pathospermia according to WHO criteria [9] at baseline sperm analysis. On the day of IUI, 97 (14.8%) had a TPMSC < 5 mil, 133 (20.3%) between 5 and 15 mil, and 425 (64.9%) > 15 mil.

**Table 1:** Patient characteristics at first successful or last ineffective IUI cycle

	Overall	HCG		*p*	Missing (%)
		Negative	Positive		
Number of patients	758	611	147		
Age (mean (SD))	33.4 (5.5)	33.6 (5.6)	32.1 (4.9)	0.003	0.0
BMI (median [IQR])	22.3 [20.5, 25.8]	22.1 [20.4, 25.5]	23.0 [20.9, 26.5]	0.064	7.8
Reasons for infertility (%)					
Female only	255 (33.6)	204 (33.4)	51 (34.7)	0.727	
Male only	152 (20.1)	122 (20.0)	30 (20.4)		
Female and male	154 (20.3)	129 (21.1)	25 (17.0)		
Idiopathic	197 (26.0)	156 (25.5)	41 (27.9)		
Female infertility diagnosis (%)				0.015	6.2
No known reason	349 (49.1)	278 (48.8)	71 (50.4)		
Anovulatory	191 (26.9)	141 (24.7)	50 (35.5)		
Endometriosis	74 (10.4)	66 (11.6)	8 (5.7)		
Tubal obstruction	58 (8.2)	50 (8.8)	8 (5.7)		
Anatomical alterations	39 (5.5)	35 (6.1)	4 (2.8)		
AMH (median [IQR])	2.4 [1.0, 5.1]	2.3 [0.9, 4.9]	3.1 [1.6, 6.2]	< 0.001	14.4
AMH (%)					
< 1 ng/ml	160 (24.7)	143 (27.8)	17 (12.7)	< 0.001	
≥ 1 ng/ml	489 (75.3)	372 (72.2)	117 (87.3)		
TPMSC (median [IQR])	24.0 [9.8, 51.8]	23.6 [8.4, 51.4]	28.2 [15.9, 54.9]	0.061	13.6
TPMSC (%)					
< 5 mil	97 (14.8)	86 (16.5)	11 (8.2)		
5–15 mil	133 (20.3)	112 (21.5)	21 (15.7)		
≥ 15 mil	425 (64.9)	323 (62.0)	102 (76.1)		
Stimulating agent (%)				0.157	16.1
Gonadotropin	325 (51.1)	256 (50.7)	69 (52.7)		
Clomifen	172 (27.0)	144 (28.5)	28 (21.4)		
Gonadotrop. + Clomifen	5 (0.8)	5 (1.0)	0 (0.0)		
None	134 (21.1)	100 (19.8)	34 (26.0)		
Number of cycles (%)				0.101	0.0
One	342 (45.1)	265 (43.4)	77 (52.4)		
Two	241 (31.8)	195 (31.9)	46 (31.3)		
Three	119 (15.7)	101 (16.5)	18 (12.2)		
Four or more	56 (7.4)	50 (8.2)	6 (4.1)		

*AMH* anti-Mullerian hormone, *BMI* body mass index, *HCG* human chorionic gonadotropin, *IQR* interquartile range, *SD* standard deviation, *TPMSC* total progressive motile sperm count

Among included IUI cycles, 10.9% resulted in a pregnancy and 19.4% of couples achieved pregnancy after one or more treatment attempts. Most couples had one to three cycles, with only 56 (7.4%) undergoing four or more cycles before achieving pregnancy or discontinuing IUI treatment (Table 2). As a result, we observed only six pregnancies in women with four or more IUI cycles. Rather than a decrease in the per cycle success rate, this seems to reflect the low number of patients continuing beyond three cycles. In fact, the number of observed pregnancies for each cycle lay within the 5% and 95% percentiles of expected outcomes (Table 2) and Fisher’s exact test indicated no variation in cycle success rate (*p* = 0.673). Successful treatment with IUI was statistically significantly associated with lower female age, infertility due to anovulation or unknown female infertility reason, higher levels of AMH and TPMSC > 15 mil (Table 1). The use of different medications (clomiphene, gonadotropins) for ovarian stimulation showed no significant influence on pregnancy rates.

**Table 2:** Number and proportion of patients and pregnancies per IUI cycle

Cycle	1	2	3	4	5	6	7	8	9
Number of patients	758	416	175	56	16	6	5	3	2
% of patients	100.0	54.9	23.1	7.4	2.1	0.8	0.7	0.4	0.3
Number of pregnancies	77	46	18	5	0	0	0	0	1
% of pregnancies	10.2	11.1	10.3	8.9	0.0	0.0	0.0	0.0	50.0
Cumulative % of pregnancies	10.2	16.2	18.6	19.3	19.3	19.3	19.3	19.3	19.4
Expected number of pregnancies									
5% percentile	62	31	10	2	0	0	0	0	0
95% percentile	94	55	26	10	4	2	2	2	1

Percentiles were calculated based on a binomial distribution with a constant pregnancy rate of 10.9%

### Multivariable analysis

In multivariable analysis, AMH levels ≤ 1 ng/ml (OR 0.50, 95% CI 0.29–0.87, *p* = 0.014), TPMSC ≤ 5 mil (OR 0.37, 95% CI 0.19–0.723, *p* = 0.004), and a diagnosis of endometriosis, uterine malformation, or tubal obstruction (OR 0.54, 95% CI 0.33–0.89, *p* = 0.016) were associated with a decreased per cycle success rate (Table 3: Model 3). Furthermore, TPMSC between 5 and 15 mil (OR 0.61, 95% 0.37–1.0, *p* = 0.049) and age ≥ 35 years (OR 0.63, 95% CI 0.41–0.97, *p* = 0.034) were moderately associated with lower per cycle success rates. A model using age, TPMSC and female infertility diagnosis (Model 1) was preferred based on the c-index whereas a model adding female age (Model 2) was preferred based on AIC. BIC was similar for Model 1 and 2. Model 2’s c-index was slightly lower but not statistically significant different due to wide confidence intervals. A model including all candidate variables showed no further improvement of discriminative power. Based on a combination of AIC, BIC, and c-index, Model 2 was chosen for the development of our clinical score.

**Table 3:** Multivariable associations with per cycle pregnancy rate and model fit for three increasingly complex continuation ratio models

	Model 1		Model 2		Model 3	
	OR (95% CI)	*p*	OR (95% CI)	*p*	OR (95% CI)	*p*
Base odds	0.21 (0.14–0.30)		0.22 (0.15–0.33)		0.21 (0.15–0.32)	
Cycle number	0.97 (0.82–1.15)	0.718	0.97 (0.82–1.15)	0.718	0.97 (0.82–1.15)	0.749
Age						
< 35 years	1		1		1	
≥ 35 years	0.53 (0.36–0.77)	< 0.001	0.65 (0.43–0.97)	0.034	0.65 (0.43–0.97)	0.037
TPMSC						
< 5 mil	0.40 (0.21–0.76)	0.006	0.38 (0.20–0.74)	0.004	0.38 (0.20–0.73)	0.004
5–15 mil	0.63 (0.38–1.02)	0.060	0.62 (0.38–1.00)	0.052	0.62 (0.38–1.01)	0.055
≥ 15 mil	1		1		1	
Female infertility diagnosis						
No known infertility/anovulatory disorder	1		1		1	
Other	0.52 (0.32–0.86)	0.010	0.53 (0.33–0.88)	0.013	0.54 (0.33–0.89)	0.016
AMH						
< 1 ng/ml			0.50 (0.29–0.88)	0.016	0.50 (0.29–0.87)	0.014
≥ 1 ng/ml			1		1	
BMI						
< 30 kg/m^2^					1	
≥ 30 kg/m^2^					1.32 (0.78–2.24)	0.308
AIC	929.3		923.6		924.5	
BIC	960.9		960.5		966.6	
c-index	0.76 (0.71–0.81)		0.73 (0.68–0.78)		0.72 (0.67–0.77)	

*AIC* Akaike Information Criterion, *AMH* anti-Mullerian hormone, *BIC* Bayesian Information Criterion, *BMI* body mass index, *HCG* human chorionic gonadotropin, *IQR* interquartile range, *SD* standard deviation, *TPMSC *total progressive motile sperm count

### Clinical score

**Fig. 1:**
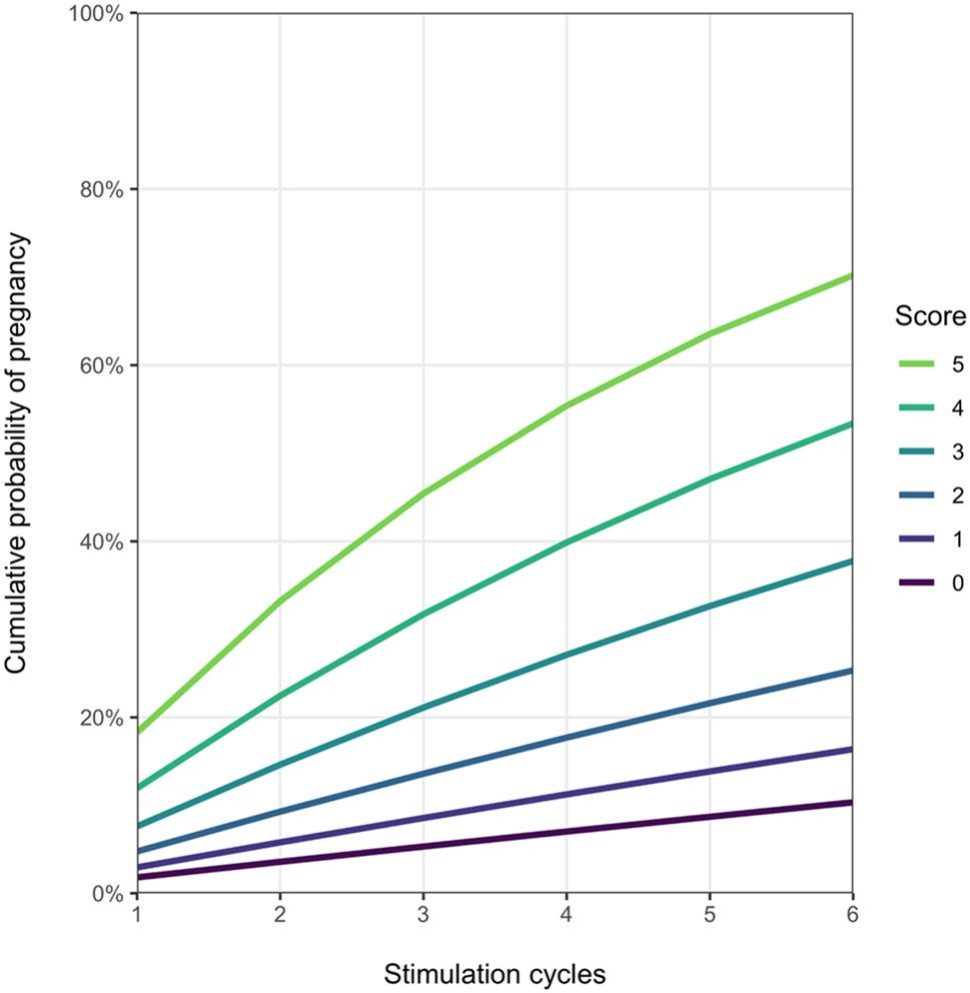
Calculation of proposed IUI success score (ISS) and associated cumulative probabilities of achieving pregnancy

The scoring system derived from the multivariable analysis assigns between 0 and 2 points for female age, AMH, female infertility diagnosis and TPMSC, respectively (Fig. 1). The sum of points results in a score between 0 and 5, which we called “IUI success score”. The probability of achieving pregnancy per cycle and cumulatively increases with higher score. For example, a woman of 28 years with an AMH of 4,65 ng/ml, PCOS and a partner having a TPMSC of 17 mil, would be assigned 1, 1, 1 and 2 points, respectively, resulting in an IUI success score of 5 and an estimated probability of ~ 45% to achieve pregnancy after three IUI cycles. A different woman of 33 years with an AMH of 1,21 ng/ml, a history of mild endometriosis and a partner with TPMSC of 3 mil would receive 1, 1, 0 and 0 points, adding up to a score of 2. After three IUI cycles, this couple has an estimated probability of ~ 10%, and even after 6 cycles they would reach only a probability of 25% based on extrapolated data. Applying the score to our cohort, 4 couples (0.5%) received a score of 0, 21 a score of 1 (2.7%), 108 a score of 2 (14.3%), 205 a score of 3 (27.0%), 223 a score of 4 (29.4%) and 198 a score of 5 (26.1%).

## Discussion

In this study, we were able to show that while the pregnancy rate per IUI cycle was relatively low (11%), repeated cycles led to an average overall success rate of approximately 19%. Per cycle and cumulative pregnancy rates varied considerably by couples’ characteristics. Among female factors, younger age and higher AMH were positively associated with pregnancy. Additionally, we found that women with infertility due to anovulation and those with unknown female reason for infertility had higher pregnancy rates compared to women with endometriosis, unilateral tubal factor, or anatomical alterations. TPMSC at insemination also correlated with IUI success. Based on these findings, we propose a clinical scoring system to predict a couple’s probability of achieving pregnancy. Couples are assigned 0, 1 or 2 points for each prognostic factor, adding up to a total score ranging from 0 to 5. After 3 cycles, couples in our cohort with a score of 5 had a cumulative probability of pregnancy of approximately 45%. In contrast, couples with a score of 0 only had a cumulative probability of 5% Our per cycle pregnancy rate agreed with previous studies, which reported rates between 7 and 15% [19–23]. However, our average per patient pregnancy rate was lower than the 29–43% reported by the same authors [21, 23, 24]. This might have been due to a lower number of IUI cycles per couple in our study. On average, only 1.9 cycles were performed in our cohort compared to 2.4–2.9 cycles in previous studies. An insufficient number of treatment cycles has been shown to reduce the per patient pregnancy rate significantly [25].

Only few couples in our cohort underwent 4 or more IUI cycles and we observed only a handful of pregnancies following the 4th cycle. This seemed to reflect the low number of patients continuing past 3 cycles rather than a genuine decrease in per cycle success rate. This is in line with the results of a recent study by Muthigi et al., reporting consistent pregnancy rates up to 6 total IUI cycles [26]. In contrast, other previous studies described a plateau of IUI success rates after 3 or 4 cycles. Khalil et al. reported only marginal increases in the cumulative birth rate after 4 cycles. However, their analysis did not account for a similar decrease in the number of patients that underwent 4 or more cycles [20]. Aboulghar et al. reported a drastically decreasing pregnancy rate in cycles 4–6 (5.6%) compared to the first 3 cycles (16.4%) [27]. While the authors correctly accounted for decreasing patient numbers, their reported success rates were unusually high in early cycles and particularly low in later cycles, compared to the wider literature [20, 21, 23, 24]. Nevertheless, it remains possible that there is a small selection effect as couples with higher chances get pregnant early and drop out. Based on our data, we believe that this effect may be less pronounced than previously reported and may be partially compensated by a detailed selection of couples considered for IUI treatment.

We found four main predictive factors that should be taken into account when counseling infertility patients regarding IUI: female age, AMH, female infertility diagnosis and TPMSC. It has long been known that female fertility declines with advancing age due to a decrease both in oocyte quantity and quality [28]. Accordingly, lower IUI success rates at increasing female age have been reported by several previous studies [19, 21, 23, 24, 29–33]. Less clear is the association between IUI success rates and AMH. This hormone has been proven to be a reliable marker of ovarian reserve [34, 35], typically declining at increasing age [36, 37]. It is, therefore, difficult to understand to what extend the observed effects of AMH are biased by age. However, AMH seems to reflect the decreasing number of oocytes more than their quality. In fact, Morin et al. [38] were able to show equivalent live birth rates per euploid embryo transfer in women with diminished ovarian reserve undergoing IVF as compared to age-matched controls, indicating that the pathophysiological mechanisms mediating a decline in oocyte quantity may differ from the ones leading to poor oocyte quality. Regarding IUI, the results reported in the literature are still conflicting, some authors describing AMH as good predictor of pregnancy rates [32, 39–41], while other studies don’t find any difference in IUI success rates based on AMH [42–45]. Among these, a recent retrospective cohort study showed no statistically significant difference of live birth rates in patients < 35 years with AMH < 1.0 ng/ml undergoing IUI compared to age-matched controls with AMH ≥ 1.0 ng/ml  [45]. However, their data still showed a tendency versus lower conception rates in the group with low AMH. When considering only the first IUI cycle performed per couple—as it was done in the cited study—in our cohort, we still observed lower pregnancy rates in the group of patients < 35 years with AMH < 1.0 ng/ml then in the same age group with higher AMH. In patients ≥ 35 years, this effect was less pronounced, but still present. Based on these findings and the similarity in AIC, BIC, and c-indices of Models 1 and 2, we decided to include AMH in our predictive score. In accordance with previous studies we also found higher per cycle and per patient pregnancy rates for women with unexplained infertility or anovulatory cycles compared to those with mild endometriosis, unilateral tubal factor, or anatomical alterations [20, 21, 24, 29, 46, 47]. This is clinically plausible, as endocrine disorders leading to anovulation or suboptimal follicle maturation can be overcome by ovarian stimulation used in combination with IUI, while anatomical and immunological factors cannot be compensated by IUI. Finally, sperm characteristics and more precisely TPMSC were found to be predictive of IUI success. However, due to the retrospective design, only pre-wash semen parameters were available. It has previously been reported that the minimum TPMSC for the performance of an IUI should be > 5 mil [21, 48]. Recently, Muthigi et al. found maximal pregnancy rates at IUI with a TPMSC ≥ 9 mil [26]. These data referred to TPMSC after semen preparation, whereas the correlation between pre-wash TPMSC and live birth rate after IUI was previously found to be low [49]. In contrast, in this study we observed a significant influence of pre-wash TPMSC on pregnancy rates after IUI. Future studies should further investigate this correlation, as pre-wash semen parameters are much more widely available in routine care.

Previous studies reported the number of dominant follicles as a further predictive factor for IUI success rates. Several authors described higher pregnancy rates in the presence of at least 2 preovulatory follicles [21, 24, 50]. Accordingly, the number pf preovulatory follicles is considered in all the previously published models predicting IUI success [3, 4, 6, 7]. However, in recent years multiple pregnancies have become a rising concern. It has been shown that strict cancelation policies in case of multifollicular growth are effective in reducing the risk of multiple gestations following IUI [10–12]. These findings limit the applicability of the cited predictive models. Based on our national guidelines [51], our institute cancels all cycles with recruitment of > 2 follicles > 16 mm. We, therefore, a priori excluded the number of leading follicles from our IUI success score. In an additional univariate sensitivity analysis (not shown), pregnancy rate was not significantly influenced by the number of follicles. Subgroup analysis showed a significant difference between the average number of leading follicles in successful versus unsuccessful IUI cycles only for patients with endometriosis (unsuccessful IUI cycle: 1.1 ± 0.4 leading follicles, successful IUI cycle: 1.5 ± 0.5; *p* = 0.041). It is possible that in case of mild endometriosis the presence of more than 1 leading follicle at OI and IUI increases the chances of pregnancy. This is plausible as endometriosis has been reported to be associated with reduced oocyte quality [52]. However, the number of patients with endometriosis in our cohort was very small (*n* = 74), limiting the interpretability of this finding.

While previous studies found higher pregnancy rates when IUI was preceded by ovarian stimulation with gonadotropins compared to clomiphene or letrozole [3, 4, 53], we didn’t observe significant differences in IUI success based on stimulation protocol. This might be related to differences in the study population, as different stimulating agents have been shown to be superior depending on female infertility diagnosis [12, 53–55]. Moreover, previous studies reporting better IUI outcomes with gonadotropins did not control for the number of preovulatory follicles [53]. This is important as -depending on their dosage—gonadotropins can lead to multifollicular growth more easily, possibly introducing bias into this finding.

### Strengths and limitations

Our study has several strengths. First, we report on a large clinical population undergoing routine care which reflects the population seen in most infertility centers. Second, our analysis covered a period of multiple years, adding stability to our findings. Most previously developed models predicting IUI success emphasized higher pregnancy rates with multifollicular growth. As the latest guidelines and trials [10–12, 51, 56] recommend avoiding multifollicular recruitment in order to avoid multiple gestations, these findings may be outdated. Due to the strict cancelation policy that has long been implemented at our institute, our IUI success score offers guidance in line with these updated recommendations with ovulation induction of a maximum of 2 leading follicles.

Limitations of our study include the retrospective data collection and potential unknown confounders inherent to this study type. While treatment was recommended to couples based on internal guidelines, the ultimate decision to proceed with IUI and the number of cycles was determined by the couple. We were unable to account for potential selection biases due to the type of treatment chosen or due to continuation of treatment. If couples with a low probability of pregnancy were to drop out earlier than those with a high probability, our results might be biased towards success. However, we did not observe a notable change in patient characteristics across cycles 1–5, and only few patients remained thereafter. It is, therefore, unlikely that selective drop-out had a strong influence on our results. Factors that influence patients’ choices for one or another therapy–such as financial incentives–may differ by infertility center or country and potentially limit the generalizability of our findings. Clinical variables considered for our score were only evaluated once per couple (at the first successful or the last ineffective treatment cycle, respectively). Although some variables (age, AMH, TPMSC, BMI) may change over time, it is reasonable to assume that this had only little influence on our results. The considered parameters tend to be stable in the short term [37, 57] and in almost 95% of the couples of our cohort all IUI cycles were performed within of 12 months, in 50% in consecutive menstrual cycles and therefore mainly over the course of 3–4 months. Finally, since we retrospectively extracted our data from routine patient records, we encountered occasional missing data in several of our variables. It is plausible that missingness was at random and we employed multiple imputation to mitigate the impact of missing data on our results.

### Conclusion

We showed that IUI success rates vary widely depending on couples’ characteristics and propose an easily applicable clinical tool to estimate a couple’s chance of achieving pregnancy via IUI. After external validation proving its generalizability, this model could facilitate patient-oriented counseling and decision-making.

## Data Availability

The data underlying this article will be shared on reasonable request to the corresponding author.

## Code availability

All code used for the analysis can be found at https://github.com/prockenschaub/IUI.
